# Low molecular weight ε-caprolactone-*p*-coumaric acid copolymers as potential biomaterials for skin regeneration applications

**DOI:** 10.1371/journal.pone.0214956

**Published:** 2019-04-08

**Authors:** Marco Contardi, Alejandro Alfaro-Pulido, Pasquale Picone, Susana Guzman-Puyol, Luca Goldoni, José J. Benítez, Antonio Heredia, Markus J. Barthel, Luca Ceseracciu, Giovanni Cusimano, Ornella Roberta Brancato, Marta Di Carlo, Athanassia Athanassiou, José A. Heredia-Guerrero

**Affiliations:** 1 Smart Materials, Istituto Italiano di Tecnologia, Genova, Italy; 2 DIBRIS, University of Genoa, Genoa, Italy; 3 Istituto di Biomedicina ed Immunologia Molecolare "A. Monroy", CNR, Palermo, Italy; 4 Analytical Chemistry Facility, Istituto Italiano di Tecnologia, Genova, Italy; 5 Instituto de Ciencia de Materiales de Sevilla, Centro mixto CSIC-Universidad de Sevilla, Isla de la Cartuja, Sevilla, Spain; 6 Instituto de Hortofruticultura Subtropical y Mediterránea (IHSM), La Mayora Universidad de Málaga-CSIC Algarrobo-Costa, Málaga, Spain; 7 Departamento de Biología Molecular y Bioquímica, Universidad de Málaga, Málaga, Spain; 8 Nanomaterials for Biomedical Applications, Istituto Italiano di Tecnologia, Genova, Italy; 9 Materials Characterization Facility, Istituto Italiano di Tecnologia, Genova, Italy; University of Hyderabad, INDIA

## Abstract

ε-caprolactone-*p*-coumaric acid copolymers at different mole ratios (ε-caprolactone:*p*-coumaric acid 1:0, 10:1, 8:1, 6:1, 4:1, and 2:1) were synthesized by melt-polycondensation and using 4-dodecylbenzene sulfonic acid as catalyst. Chemical analysis by NMR and GPC showed that copolyesters were formed with decreasing molecular weight as *p*-coumaric acid content was increased. Physical characteristics, such as thermal and mechanical properties, as well as water uptake and water permeability, depended on the mole fraction of *p*-coumaric acid. The *p*-coumarate repetitive units increased the antioxidant capacity of the copolymers, showing antibacterial activity against the common pathogen *Escherichia coli*. In addition, all the synthesized copolyesters, except the one with the highest concentration of the phenolic acid, were cytocompatible and hemocompatible, thus becoming potentially useful for skin regeneration applications.

## Introduction

Biodegradable, bioresorbable, and biocompatible polymers are becoming common materials for pharmaceutical uses, mainly in the design of novel medical devices such as implants, scaffolds, films, nanoparticles or nanofibers that can control the release of drugs and/or reproduce, mimic or replace some parts of the body [1–3]. Among these polymers, poly-ε-caprolactone (PCL), after a period of unpopularity respect to other resorbable polymers such as polylactides and polyglycolides [4], has regained much interest due to its excellent biocompatibility, easy and efficient synthesis, and low degradation rate in water [1, 5]. PCL is a petroleum-based polyester synthesized via high-yield lactone ring-opening polymerization (ROP) of ε-caprolactone (common name of the hexano-6-lactone) and whose final molecular weight can be strongly affected by choice of the catalyst used [6, 7]. Moreover, the molecular weight, usually between 3000 and 80000 g/mol, influences the degradation rate of PCL and can make it suitable for different applications [5, 8]. Thus, high molecular weight PCL is required for implants and scaffolds in order to maintain the stability of the material for months or years. For instance, Pitt and Schinder [9] demonstrated that PCL capsules (with a Mw ~66000 g/mol) remained intact after 2-years of implantation in rats.

On the other hand, in the cases of sutures or wound dressings, a well-designed material should be able to be resorbed by the skin in 2–3 weeks, in order to avoid non-proper healing of the wound [10]. In this sense, Albertsson *et al*. [11] reported an *in vitro* degradation and absorption of PCL powder (Mn ~3000 g/mol) by macrophage and giant cells for a period of 13 days, suggesting that low molecular weight PCL can be effective for skin regeneration applications. PCL has a melting point around 70°C, which allows its use as a biopolymer “ink” for fused deposition 3D printing of scaffolds for tissue engineering [12, 13]. In addition, Engelberg *et al*. [14] have studied the mechanical properties of pure PCL, highlighting its suitable performance for several biomedical applications such as wound dressing, surgical sutures, contraception, and dentistry [5]. Despite its versatile behavior, PCL, similarly to other synthetic polymers such as polyvinylpyrrolidone (PVP), does not show any intrinsically biological activity [15]. For this reason, these macromolecules are often blended with bioactive polymers [16–18] and agents able to promote cell attachment [19], ensure disinfection or reduce inflammatory and oxidative events [15, 20–22]. Furthermore, by exploiting the presence of the ester group, a variety of block copolymers between PCL and chitosan [23], alginate [24] and hyaluronic acid [25] have been synthesized.

To design current sutures or wound dressings, a successful strategy is the use of natural bioactive agents for partial or complete replacement of common and widely abused antibiotics and anti-inflammatories drugs [26–30]. Among them, polyphenolic compounds such as ferulic acid, *p*-coumaric acid (PCA), and curcumin, appeared as the most promising ones [29, 31]. In fact, these molecules affect several components of the inflammatory response [32, 33], ensure a strong scavenging effect against free radicals [34], and show a certain degree of antibacterial activity [35]. These molecules, in particular *p*-coumaric acid, have been chemically combined with PCL to synthesize copolymers that merge the properties of polyphenols and polycaprolactone. For example, recently, Nguyen *et al*. [36] copolymerized ε-caprolactone with different derivatives of syringic, ferulic, and *p*-coumaric acids by using Sb_2_O_3_ as a catalyst, targeting to improve the thermal characteristics of pure PCL and to replace non-biodegradable petroleum-based plastics. Instead, Li *et al*. [37] synthesized a poly(ε-caprolactone)-co-poly(4-hydroxycinnamic acid) copolymer by catalysis with stannous octanoate for use in biomedical, drug-controlled release and fluorescent probe fields. Interestingly, in both cases, an important reduction of the molecular weight was reported when PCA participated in the polymerization.

In this work, we present an alternative synthesis of ε-caprolactone-*p*-coumaric acid copolymers at different molar ratios by melt-polycondensation catalyzed by 4-dodecylbenzenesulfonic acid (DBSA) for potential biomedical applications such as wound dressing and sutures. The resultant copolymers were chemically and physically characterized and compared to pure PCL. In addition, the antioxidant and antibacterial performance, as well as the biocompatibility and hemocompatibility, were determined.

## Materials and methods

### Materials

ε-caprolactone (ε-CL, purity: 97%, MW = 114.14 g/mol), *p*-coumaric acid (PCA, purity: ≥98.0%, MW = 164.16 g/mol), and 4-dodecylbenzenesulfonic acid (DBSA, purity: ≥95%, MW = 326.49 g/mol) were purchased from Sigma-Aldrich and used without further purification.

### Polymer synthesis

**Fig 1:**
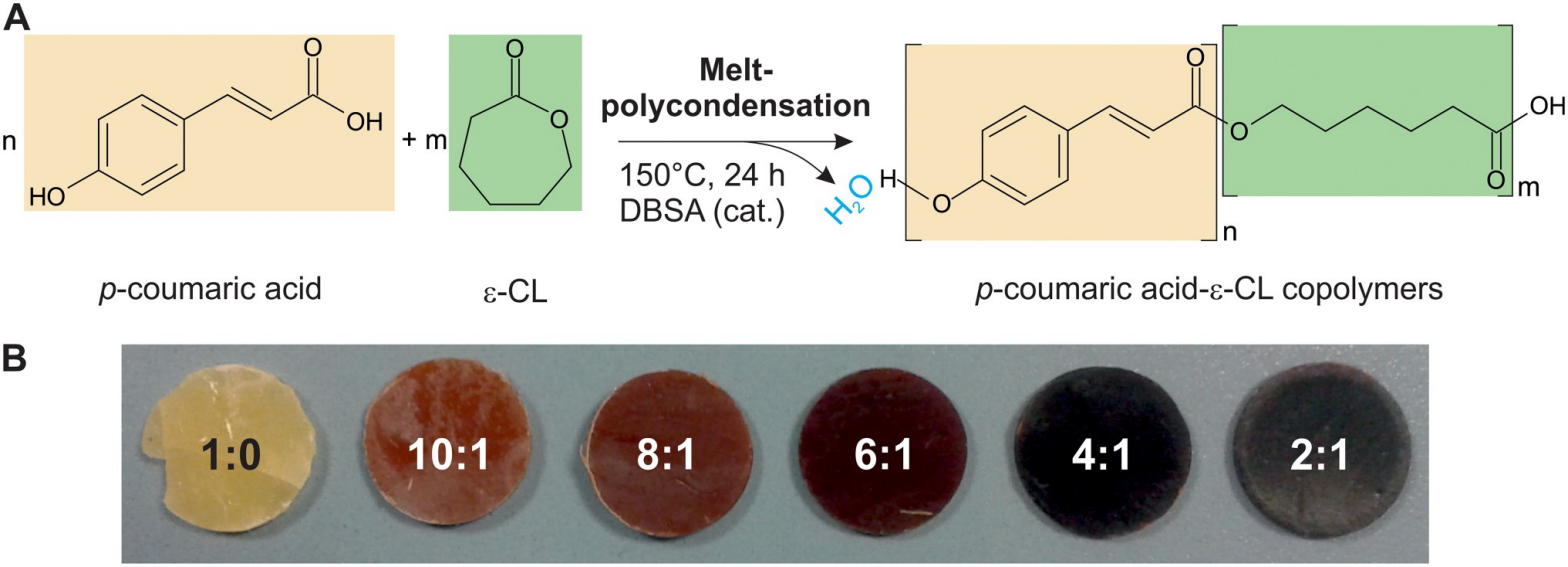
Scheme of synthesis and photographs of the biopolymers. (A) Polymerization reaction between ε-caprolactone and *p*-coumaric acid. ε-CL and PCA monomers and their corresponding repetitive units in the polyester have been highlighted in light green and light orange, respectively. (B) Photographs of the ε-caprolactone-*p*-coumaric acid copolymer films.

The synthesis of the ε-caprolactone-*p*-coumaric acid copolymers at different molar ratios (ε-CL:PCA 1:0, 10:1, 8:1, 6:1, 4:1, and 2:1) was carried out by melt-polycondensation catalyzed by DBSA, as summarized in Fig 1A. ε-caprolactone (5 mL) was heated at 150°C for 1 h. Then, different amounts of *p*-coumaric acid (0, 0.74, 0.93, 1.23, 1.85, and 3.70 g) and 10 μL of DBSA were added and the blends stirred at 150°C for 24 h. After cooling at room temperature, solids obtained were solubilized in 25 mL of chloroform and the copolyesters were precipitated with an excess of cold methanol. Samples were washed three times with water and methanol (50 mL per gram of film) and dried in vacuum for 24 h at room temperature. To form homogeneous films, copolymers were melted at 75°C for few minutes in Teflon Petri dishes and cooled at room temperature. The appearance of the films is shown in Fig 1B and sample composition and labeling are summarized in Table 1.

**Table 1:** Label, molar ratio, mole fraction, and weight % of the ε-caprolactone-*p*-coumaric acid copolymer prepared.

Label	Molar ratio		Mole fraction		Weight %	
	ε-CL	PCA	ε-CL	PCA	ε-CL	PCA
PCL/PCA 1:0	1	0	1.00	0.00	100.0	0.0
PCL/PCA 10:1	10	1	0.91	0.09	87.4	12.6
PCL/PCA 8:1	8	1	0.89	0.11	84.8	15.2
PCL/PCA 6:1	6	1	0.86	0.14	80.7	19.3
PCL/PCA 4:1	4	1	0.80	0.20	73.6	26.4
PCL/PCA 2:1	2	1	0.67	0.33	58.2	41.8

### Nuclear Magnetic Resonance (NMR) spectroscopy

For Nuclear Magnetic Resonance (NMR) characterization, ~7–8 mg of compounds were dissolved in 1 mL of THF-*d*8. NMR experiments were performed on a Bruker Avance III 400 MHz spectrometer equipped with a Broad Band Inverse (BBI) probe, without the spinning. 5 mm tubes filled with 500 μL of the sample solutions were employed. Before the acquisition, automatic matching and tuning were finely regulated, homogeneity automatically adjusted, and temperature actively controlled at 300K. The ^1^H 90° pulse was optimized by an automatic pulse calculation routine [38]. In ^1^H-NMR experiments, 45 k complex data points for 8 transients were accumulated, after a 90° flip angle, over a spectral width of 18.02 ppm (offset 6.18 ppm), at a fixed receiver gain (1), using 30 s of relaxation delay and no steady state scans. Spectra were manually phased and automatically baseline corrected. An exponential line broadening equivalent to 0.1 Hz was applied to FIDs before Fourier Transform. The ^1^H-^1^H COSY(COrrelation SpectroscopY) was performed with 1 FID, 2048 data point and 128 increment, over a spectral width of 8.93 ppm (offset at 4.17 ppm), while the ^1^H-^13^C HSQC (multiplicity edited Heteronuclear Single Quantum Coherence) was acquired with 2 FIDs, 1024 data points and 256 increments, over a spectral width of 8.93 ppm at the ^1^H and 165 ppm at the ^13^C (offset at 4.14 and 75.0 ppm, respectively). All spectra were referred to non-deuterated THF residue peak at 3.58 ppm (^1^H) and at 67.2 ppm (^13^C), according to Fulmer *et al*. [39].

*p*-coumaric acid: ^1^H NMR (THF-*d*8, 400 MHz): 12.00–7.80 br s, (2H, OH and COOH); 7.55 ppm d, *J* = 16.0 Hz (1H); 7.42 pseudo d, *J* = 8.6 Hz (2H, AA’MM’ system); 6.75 pseudo d, *J* = 8.6 Hz (2H, AA’MM’ system), 6.25 ppm d, *J* = 16.0 Hz (1H).

### Gel Permeation Chromatography (GPC)

GPC measurements were carried out using an Agilent 1260 Infinity quaternary LC system using two PLGel 5μm MIXED-C columns at 25 °C and a refractive index detector. Tetrahydrofuran (THF) was used to dissolve the samples and as eluent at 1 mL/min flow rate. The system was calibrated with Agilent EasyVial PS standards.

### Thermal characterization

The thermal degradation behavior of the samples was investigated by thermogravimetric analysis (TGA) method, using a TGA Q500 from TA Instruments. Measurements were performed using 3–5 mg of sample in an aluminum pan under inert N_2_ flow (50 mL/min) in a temperature range from 30 to 600 °C with a heating rate of 5 °C/min. The weight loss and its first derivative were recorded simultaneously as a function of time/temperature.

Differential Scanning Calorimetry (DSC) thermograms were acquired with a DSC Q20 (TA Instruments) from 30 to 150 °C under nitrogen flow (50 mL/min) at 20 °C/min by using non-hermetic aluminum pans. About 4 mg (weighted with ±0.01 mg precision) of sample were used. Specimens were first heated from 30 to 150 °C to release moisture, cooled to 30 °C and finally ramped to 150 °C.

### Mechanical tests

The mechanical properties of the PVP/PCA copolymers were determined by uniaxial tension tests on a dual column universal testing machine (Instron 3365). Films were cut in dog bone specimens (at least seven of them for each sample) with a width of 4 mm and an effective length of 25 mm. Displacement was applied at a rate of 10 mm/min. The Young’s modulus, stress at maximum load, and elongation at break were calculated from the stress-strain curves. All the stress-strain curves were recorded at 25 °C and 44% R.H.

### Water uptake

The water uptake capacity of the samples was obtained as follows: dry samples were weighed (~80 mg) on a sensitive electronic balance and placed in different chambers with controlled humidity. The humidity conditions were: 0%, 11%, 44%, 84%, 100%. After conditioning in different humidity chambers until equilibrium conditions (usually 24 h), each film was weighed, and the amount of adsorbed water was calculated based on the difference between the weight of each film and its initial dry weight.

### Water vapor permeability

The water vapor transmission rate (WVTR) and water vapor permeability (WVP) of the samples were determined at 25 °C and under 100% relative humidity gradient (ΔRH%) according to the ASTM E96 standard method. In this test, permeation chambers with a deposit (7 mm diameter and 10 mm deep) were used and filled with 400 μL of deionized water (which generates an internal 100% RH) [40]. Samples were cut in circles and mounted by sealing the top of the water deposit. The permeation chambers were placed inside a desiccator with anhydrous silica gel providing 0% RH. The mass of water transferred through the film was determined by the weight change of the permeation chamber every hour during a period of 8 hours using an electronic balance (0.0001 g accuracy). The mass loss was plotted as a function of time and the slope of each curve was calculated by linear regression. Then, the water vapor transmission rate (WVTR) was determined as below [41]:

$$WVTR(g{\left(m^{2}d\right)}^{-1})=\frac{Slope}{Area\;of\;the\;sample}$$

The WVP of the samples were then calculated as below:

$$WVP(g{\left(m\;d\;Pa\right)}^{-1})=\frac{WVTR\times L\times 100}{p_{s}\times \Delta RH}$$

where L (m) is the thickness of the sample, measured with a micrometer with a 0.001 mm accuracy; ΔRH (%) is the percentage relative humidity gradient, and p_s_ (Pa) is the saturation water vapor pressure at 25 °C [42, 43].

WVTR and WVP measurements were replicated three times for each film.

### DPPH^.^ free radical cation scavenging assay

The antioxidant capacity of the samples was determined according to the procedure described elsewhere [44]. The method is based on the scavenging of DPPH^.^ radical through the action of an antioxidant that decolorizes the DPPH^.^ solution. Briefly, 5 x 5 mm^2^ films were added to 3 mL of 0.1 mM DPPH^.^ solution in ethanol. The decrease of the absorbance was determined at 515 nm with a Cary 300 Scan UV-visible spectrophotometer at different times. All the measurements were performed in triplicates, and the results were averaged to obtain a mean value. Radical scavenging activity was expressed as the inhibition percentage of free radical by the sample and calculated as follows:

$$Radical\;Scavenging\;Activity\;(\%)=\frac{A_{0}-A_{1}}{A_{0}}\;x\;100$$

where A_0_ is the absorbance value of the control (3 mL of 0.1 mM DPPH^.^ solution in ethanol), and A_1_ is the absorbance value of the sample at different times.

### Cell culture and treatments

Primary human dermal fibroblasts (HDFa) (Thermo Fisher Scientific, Milan, Italy) were cultured with DMEM medium (Celbio, Milan, Italy) supplemented with 10% fetal bovine serum (FBS) (Gibco-Invitrogen, Milan, Italy), 2mM glutamine, 1% penicillin and 1% streptomycin (50 mg/mL). Cells were maintained in a humidified 5% CO_2_ atmosphere at 37 ± 0.1 °C. For cytocompatibility assays, HDFa cells were seeded on 96-well flat-bottom plate at a density of 4x10^3^ per well. After UV sterilization 2 mg of PCL/PCA films at a different molar ratio (*i*.*e*., 1:0, 10:1, 8:1, 6:1, 4:1, and 2:1) were plated on HDFa monolayer, and the samples were incubated at 37 °C for 72 hours.

### Determination of cell viability

Cell viability was measured by MTS assay (Promega Italia, S.r.l., Milan, Italy). MTS [3-(4,5-dimethylthiazol-2-yl)-5-(3-carboxymethoxyphenyl)-2-(4-sulphophenyl)-2H-tetrazolium] was utilized according to the manufacturer’s instructions. HDFa cells were plated in a 96-well plate, and after treatment, 20 μL of the MTS solution were added to each well and incubated for 4 h at 37 °C in a humidified incubator with 5% CO_2_. The absorbance was read at 490 nm on the Microplate reader Wallac Victor 2 1420 Multilabel Counter (PerkinElmer Inc., Monza, Italy). The results were expressed as the percentage of MTS reduction relative to the control and presented as the mean ± standard deviation (SD) and were evaluated according to the ISO10993-5 standard guidelines [45] in which toxicity for direct-contact method is scored from 0 to 4 (0 = non-cytotoxic, 1 = slightly cytotoxic, 2 = mildly cytotoxic, 3 = moderately cytotoxic and 4 = severely cytotoxic). The treated cultured cells and the controls were morphologically analyzed by microscopy inspection on an Axio Scope 2 microscope (Zeiss).

### Hemolysis assay

Hemolysis assay was performed according to the protocol of Picone *et al*. [46]. Briefly, 5 mL of venous blood collected early in the morning from a healthy donor was drawn directly into K2 EDTA coated Vacutainer tubes to prevent coagulation. After centrifugation at 500*g* for 5 min, the hematocrit and plasma level were marked on the tube. Then the plasma was removed and replaced with 150 mM NaCl, and the tube was centrifuged at 500*g* for 5 min. This step was repeated three times. After, the supernatant was replaced with PBS at pH 7.4. 200 μL of diluted (1:50) erythrocytes were pipetted into a 96-well plate. Thereafter, different PCL/PCA samples (in particular, 1:0, 6:1, 4:1, and 2:1) at 10 mg/mL or 10 μL of 20% Triton X-100, as a positive control, were added to the erythrocytes sample. The plate was incubated at 37 °C for 2 h and then centrifuged for 5 min at 500*g* to pellet whole erythrocytes. Then, 100 μL of the supernatant was transferred from each well into a clear, flat-bottomed 96-well plate and absorbance, due to free hemoglobin presence, was read at 490 nm by using a plate reader (Wallac Victor 2 1420 Multilabel Counter (PerkinElmer, Inc. Monza, Italy). After background subtraction, the average absorbance of the positive control was determined. All experimental data points were normalized with this mean absorbance value, which represents 100% hemolysis.

### Antibacterial test

The antimicrobial activity of PCL/PCA samples was tested against *Escherichia coli* (*E*. *coli*) by using agar-disk diffusion method. Pieces of PCL/PCA films (~250 mg) with different PCA ratios (1:0, 6:1, 4:1, and 2:1) were sterilized under UV lamp at 254 nm for 30 minutes. An aliquot of *E*. *coli* o.n. culture, approximately 10^9^ CFU/mL, was diluted (1:1000) and 10 μL were spread onto the LB agar plates. After 10 minutes, the samples were placed on the plates and incubated at 37°C for 24 and 72 h. The inhibitory effect of PCL/PCA on bacteria growth was determined by assessing the dimensions of the colonies in the PCA released zone. Three replicate plates were used for each concentration of the film. For the inhibitory growth test, 100 μL of *E*. *coli* o.n. bacterial culture, approximately 10^9^ CFU/mL, was added to fresh LB medium (10 mL) with or without PCL/PCA at different molar ratios (1:0, 6:1, 4:1, 2:1). The exponential growth was determined by spectrophotometric measurements (600 nm) every 30 minutes up to 360 minutes. Data of three independent experiments were obtained.

### Statistical analysis

The significance of the differences in the mean values of groups was evaluated using the analysis of variance (one-way ANOVA). The analysis was followed by Bonferroni’s *post hoc* test. Differences were considered significant when the p-value was ≤ 0.05.

## Results and discussion

### Chemical characterization

**Fig 2:**
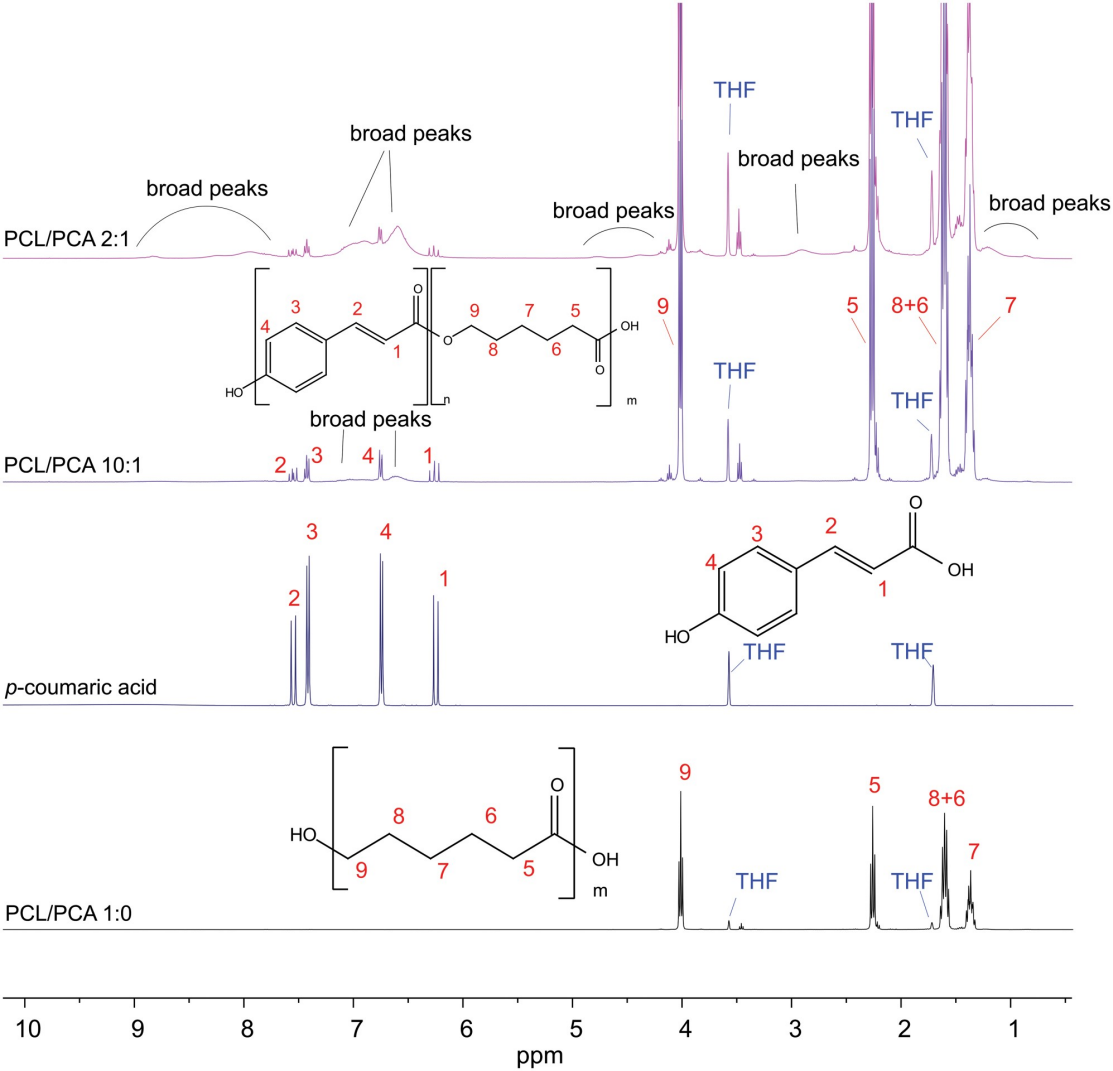
NMR characterization. ^1^H-NMR spectra of PCL/PCA 1:0, 10:1, and 2:1 and *p*-coumaric acid in THF-*d*_*8*_. Assignments are included.

The chemical characterization of the copolymers was carried out by ^1^H NMR. As representative examples, the spectra of PCL/PCA 10:1 and 2:1 as well as those of 1:0 and *p*-coumaric acid (for comparison purposes) are shown, Fig 2. PCL/PCA 1:0 showed the typical chemical shifts of polycaprolactone at 4.01, 2.26, 1.61, and 1.37 ppm, associated with the different local magnetic environment of protons of PCL (a complete assignment and characterization can be found in Fig 2 and S1 Fig). On the other hand, for *p*-coumaric acid, the chemical shifts at 7.55 and 6.24 ppm and 7.41 and 6.74 ppm were attributed to the protons of the double bond and aromatic ring groups, respectively (more details are present in the Materials and methods section, NMR part). Instead, copolymers’ spectra showed the fingerprint of the aliphatic and aromatic components. Moreover, broad peaks, whose intensity depended on the PCA content, were also observed. Such signals are characterized by long correlation times (τ_c_) which is typical for species that move slowly in solution. These broad peaks have been previously observed in other polyesters where phenolic compounds have been copolymerized with aliphatic monomers[36].

To further characterize the ε-caprolactone-*p*-coumaric acid copolymers, their molar mass was determined by GPC. Table 2 shows M_n_, M_w_, and PDI values. As observed, the participation of *p*-coumaric acid as a monomer decreased the molar mass from M_n_ = 4500 g/mol and M_w_ = 6850 g/mol for PCL/PCA 1:0 to M_n_ = 2800 g/mol and M_w_ = 3100 g/mol for PCL/PCA 2:1. This is a reduction of ~38% and ~55% for M_n_ and M_w_, respectively. Most likely, such a drop of molecular weight can be a consequence of a lower reactivity of PCA in comparison to ε-caprolactone during the melt-polycondensation. On the other hand, the polydispersity was also decreased from 1.55 for PCL/PCA 1:0 to 1.12 for PCL/PCA 2:1. In a linear step-reaction, the polydispersity index can be defined as PDI = 1 + p, where p is the conversion degree [47]. This equation predicts PDI values ranging from 1 to 2 as the conversion increases. Values listed in Table 2 can be interpreted in terms of a competition between chain growth and chain condensation reactions. In the case of PCL, the chain condensation rate is high and a broad distribution of molecular sizes is obtained (higher PDI). However, and due to diffusion limitations in the molten state, the conversion degree is low and moderate PDI and low Mw polymers are obtained. When PCA is added, the chain growth reaction is favored vs the chain condensation process and a more uniform size distribution is obtained (lower PDI values). Therefore, a low chain condensation degree causes the reduction of the average molecular weight of PCL-PCA copolymers.

**Table 2:** Number average molecular mass (M_n_), mass average molecular mass (M_w_), and polydispersity index (PDI) of PCL/PCA copolymers.

Sample	M_n_ (g/mol)	M_w_ (g/mol)	PDI
1:0	4500	6850	1.55
10:1	4200	5800	1.37
8:1	3950	5000	1.27
6:1	4000	5200	1.31
4:1	3650	4400	1.21
2:1	2800	3100	1.12

### Thermal analysis

**Fig 3:**
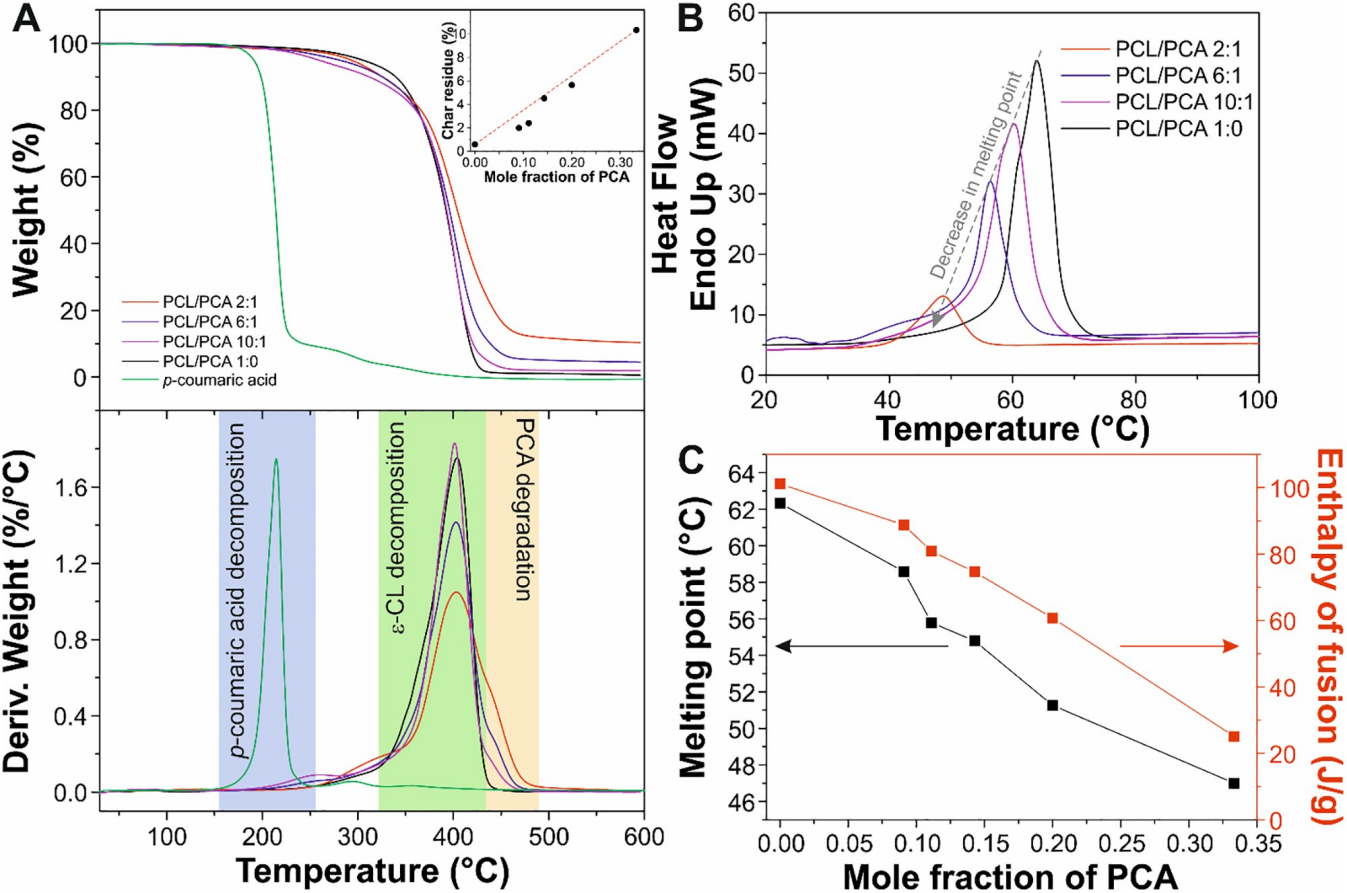
Thermal stability analysis. (A) TGA thermograms (top) and derivative thermogravimetric curves (bottom) of PCL/PCA 1:0, 10:1, 6:1, and 2:1 samples and pure *p*-coumaric acid. The inset in the top graph shows the percentage of char residue as a function of the mole fraction of PCA. (B) DSC curves of PCL/PCA 1:0, 10:1, 6:1, and 2:1 samples in the range of temperature between 20 and 100°C. (C) Melting point and enthalpy of fusion of PCL/PCA copolymers respect to the mole fraction of PCA.

The thermal behaviour of PCL/PCA samples and pure *p*-coumaric acid was investigated by TGA and DSC, Fig 3. Fig 3A shows the thermogravimetric analysis (top) and the corresponding derivative thermogravimetric curves (bottom) of the PCL/PCA 1:0, 10:1, 6:1, and 2:1 samples. PCL/PCA 1:0 presented a single weight loss at ~404 °C, while the degradation of p-coumaric acid was at ~215°C. The copolymers showed two weight losses: one at ~404°C associated with the degradation of the aliphatic fraction in the copolymer and another one, a shoulder in the derivative thermogravimetric curves, at ~445°C related to the thermal decomposition of *p*-coumarate repetitive units [48]. No degradation of free *p*-coumaric acid was observed, indicating that it was not present in the samples. Furthermore, the presence of PCA increased the char residue after TGA measurement from ~0.5% for PCL/PCA 1:0 to ~10% for PCL/PCA 2:1, inset of Fig 3A.

DSC results are reported in Fig 3B and 3C. DSC thermograms of PCL/PCA 1:0, 10:1, 6:1, and 2:1 samples are displayed in Fig 3B. A single thermal event associated with the melting point of the copolymers was observed. A decrease in melting temperatures and lower enthalpies of fusion were determined, as PCA content is raised in Fig 3C. Thus, T_m_ and ΔH_f_ ranged from ~62°C and ~101 J/g, respectively, for PCL/PCA 1:0 to ~47°C and ~25 J/g, respectively, for PCL/PCA 2:1. These decreases can be attributed to the reduction of molecular weight and to the lower interaction between polymer chains due to the different nature (*i*.*e*., aromatic and aliphatic) of both repetitive units.

### Mechanical characterization

**Fig 4:**
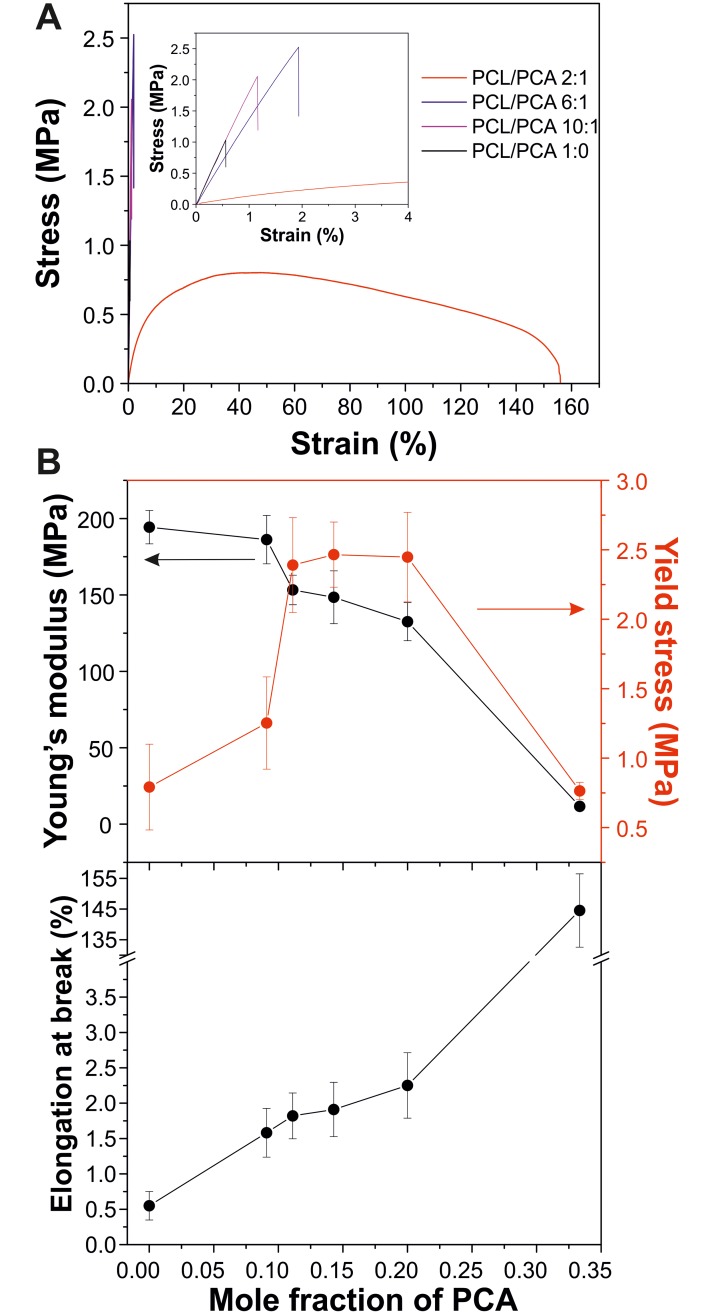
Mechanical characterization of the biopolymers. (A) Stress-strain curves of PCL/PCA 1:0, 10:1, 6:1, and 2:1 films. The inset shows a magnification at low strain values. (B) Variation of Young’s modulus, yield stress, and elongation at break as a function of the mole fraction of PCA.

Mechanical properties of the different PCL/PCA copolymers films were characterized by uniaxial tensile tests, Fig 4. Fig 4A shows the typical stress-strain curves of PCL/PCA 1:0, 10:1, 6:1, and 2:1 copolymers. As observed, most of them are brittle with high elastic moduli and low elongation at break. However, PCL/PCA 2:1 displayed a ductile behavior with a lower rigidity, a stress softening region and a much larger elongation at break. The values of Young’s modulus, yield stress, and elongation at break are plotted as a function of the mole fraction of PCA in Fig 4B. PCL/PCA 1:0 showed a Young’s modulus of 195 MPa and low values of yield stress (~0.8 MPa) and elongation at break (~0.6%). As PCA content is increased, copolymers are slightly plasticized, reducing Young’s moduli though increasing the values of stress and elongation at break, despite the lower molecular weights. For instance, PCL/PCA 4:1 showed a value of Young’s modulus, stress, and elongation at break of ~132 MPa, ~2.5 MPa, and ~2.3%, respectively. Instead, the copolyester 2:1 is very different, with a Young’s modulus of ~12 MPa, a yield point at ~0.8 MPa, and an elongation at break of ~145%. Such change can be related to a significant low molecular weight and a scarce intermolecular interaction between polymers chains, as confirmed by DSC.

### Water uptake, breathability, and antioxidant properties

**Fig 5:**
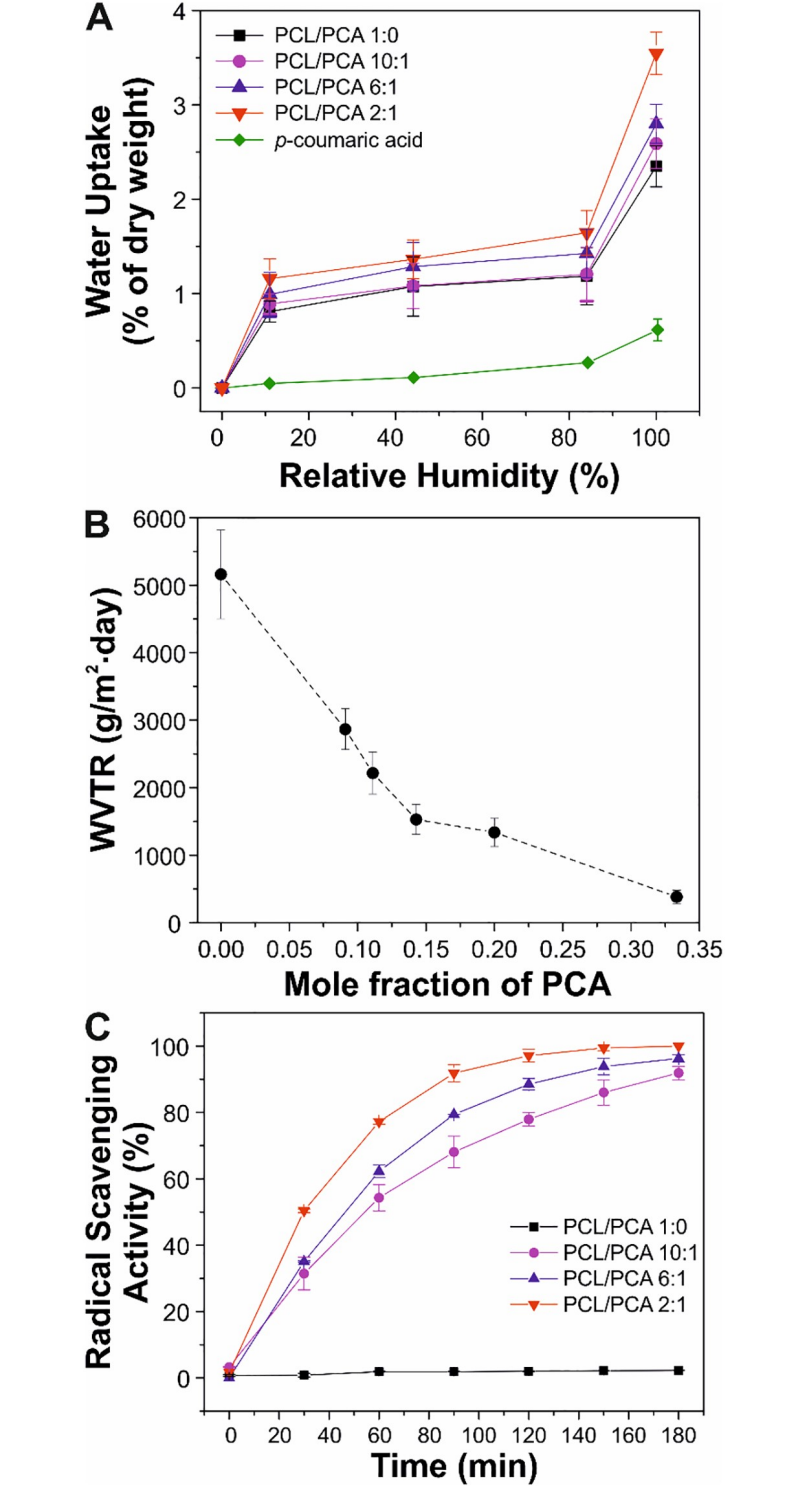
Evaluation of the water uptake, breathability, and antioxidant properties of the biopolymers. (A) Variation of the water uptake of PCL/PCA 1:0, 10:1, 6:1, and 2:1 and pure *p*-coumaric acid with the relative humidity. (B) Water vapor transmission rate (WVTR) as a function of mole fraction of PCA. (C) Antioxidant capacity of PCL/PCA 1:0, 10:1, 6:1, and 2:1 copolymers by using DPPH∙ free radical cation scavenging method.

The water uptake of PCL/PCA films was assessed, as reported in Fig 5A. A general low-absorption pattern was observed with the highest values occurring at 100% RH (~2.3% for PCL/PCA 1:0, ~2.6% for PCL/PCA 10:1, ~2.8% for PCL/PCA 6:1, and ~3.6% for PCL/PCA 2:1). The slight increase of water uptake with the PCA content can be ascribed to the lower molecular weight of the polymers that, in turn, is related to a higher presence of terminal polar functional groups, such as–OH and–COOH. On the other hand, pure p-coumaric acid showed the lowest values, most likely because of the strong interactions by H-bonds between carboxyl and hydroxyl groups of the molecules in crystalline and hydrophobic form, as recently reported in Contardi *et al*.[22]. Fig 5B shows the water vapor transmission rate (WVTR) values. WVTR decreased linearly from PCL 1:0, with a value of 5160 g m^-2^ day^-1^, to PCL/PCA 6:1, with a value of 1528 g m^-2^ day^-1^. Then, the drop was less intense for PCL/PCA 2:1, with a WVTR of 382 g m^-2^ day^-1^. In general, as recently reported by Xu *et al*.[49], materials with a WVTR between 3000 and 1000 g m^-2^ day^-1^ generate the best microenvironment conditions in the wound bed to promote the healing, thus suggesting a potential application of PCL/PCA copolymers, except for 2:1, in the field of wound healing.

In the case of chronic wounds and burns, the wound bed environment is characterized by the presence of high levels of reactive oxygen species (ROS) due to the intense inflammatory response [50]. Therefore, wound dressing materials should ensure an adequate scavenging activity in order to be able to promote the healing process by balancing the level of free radicals [34]. For this reason, DPPH∙ free radical cation scavenging assay was carried out to investigate if PCA can maintain its antioxidant properties after the copolymerization with PCL. As shown in Fig 5C, PCA-containing samples were able to scavenge almost all the free radicals in 3 hours, while PCL/PCA 1:0 films, with no PCA content, showed no antioxidant activity.

### Cytocompatibility and hemocompatibility

**Fig 6:**
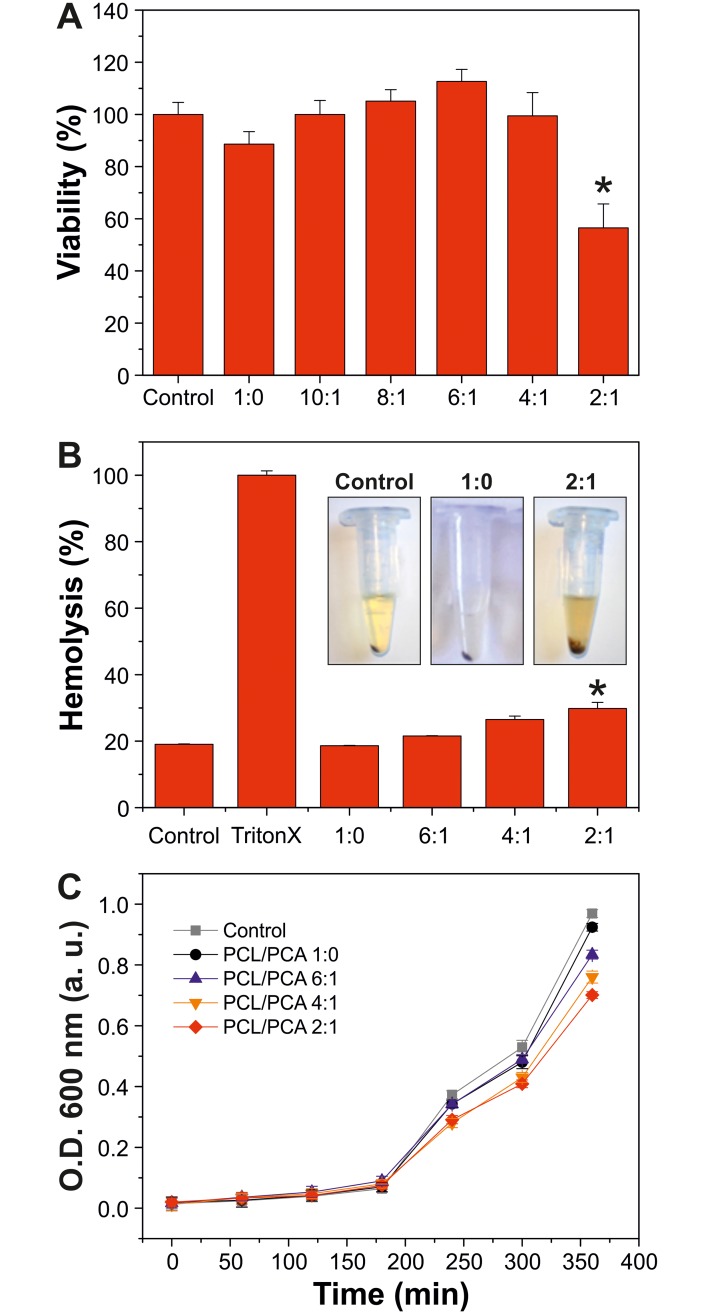
Biocompatibility, hemocompatibility and antibacterial properties of the biomaterials. (A) Histogram of MTS assay of cells incubated for 72 h with the PCL/PCA films at different mole ratios. The control sample does not present any PCL/PCA materials. *p< 0.05 *vs* control. (B) Histogram relative to the absorbance of released Hb after treatment with PCL/PCA films 1:0, 6:1, 4:1, and 2:1. The values are expressed as the percentage respect to the positive control (TritonX). *p< 0.05 *vs* control. The insets show the photographs of human erythrocytes incubated with the control and PCL/PCA 1:0 and 2:1 after centrifugation. (C) Growth curve of *E*. *coli* in the presence of PCL/PCA 1:0, 6:1, 4:1, and 2:1 films. The control sample does not present any PCL/PCA materials.

The cytotoxicity of PCL/PCA copolymers was evaluated using two different tests: the “direct-contact” method and the MTS cell viability assay, as described by Picone et al. [51]. PCL/PCA films at different mole ratios were placed on primary human dermal fibroblasts (HDFa) and incubated for 72 hours. Regarding cell morphology, and more specifically the detachment, lysis, vacuolization, and cell numbers, no changes were observed, S2 Fig, hence classifying the samples as of grade 0, except for the PCL/PCA 2:1, in which changes in shape, number and dimension were observed. On the other hand, according to the ISO10993-5 guidelines [45], a biomaterial is considered cytotoxic when its viability is below 70%. By MTS assay, it was found a minimum of 90% of cell viability for all of the samples, except for PCL/PCA 2:1 (cell viability 57%), indicating that the contact with the biomaterial, excluding the highest mole ratio, did not induce any cell toxicity or abnormal cell growth, Fig 6A.

Hemocompatibility is an index of the presence of intact or lysate erythrocytes in the blood. A good hemocompatibility is a mandatory requirement for materials in contact with blood, like in the case of surgical sutures [52, 53]. Fig 6B summarizes the main results about hemocompatibility of PCL/PCA copolymers. The values were normalized to the complete hemolysis achieved with Triton X (positive control) [51]. No hemolysis was observed both in treated and no treated (control) blood samples with values ranged between ~19% and ~27%, except for the PCL/PCA 2:1 sample in which light hemolysis was observed (hemolysis ~30%), inset of Fig 6B. This sample showed significant difference with respect to the other copolymers.

### Effect of PCL/PCA films on bacterial growth

As reported in the literature, the antibacterial activity of phenolic compounds is caused by their ability to produce irreversible permeability changes of the bacterial membrane and to interact with the DNA, affecting the replication process [35, 54]. The antibacterial activity of films of PCL/PCA 1:0 and the samples with the highest concentrations of PCA (*i*.*e*., PCL/PCA 6:1, 4:1, and 2:1) against *E*.*coli* was evaluated by the inhibition zone assay, as shown in S3 Fig. After 24 hours of growth, no inhibition zone was observed in any sample. In contrast, after 72 hours of growth, a slight decrease in the bacteria colony growth in PCL/PCA 4:1 and 2:1 was reported. To quantify the previous observation, a dilution of *E*.*coli* o.n. bacterial growth was incubated in test tubes containing LB without (control) or with PCL/PCA (1:0, 6:1, 4:1, and 2:1) copolymers. The kinetics of the bacterial growth was spectrophotometrically monitored, Fig 6C. The kinetic curves indicated that the growth was slightly reduced in the PCA-rich samples as a function of *p*-coumaric acid concentration. For instance, at 360 minutes the values of O.D. (600 nm) ranged between 0.92 for PCL/PCA 1:0 (close to the control value) to 0.70 for PCL/PCA 2:1 (*i*.*e*., a reduction of ~24%).

## Conclusions

The physicochemical properties of ε-caprolactone-*p*-coumaric acid copolymers can be tuned by controlling the relative proportions of both monomers. These molecules can polymerize by melt-polycondensation catalyzed by the sulfonic acid DBSA, originating thermoplastic free-standing polyester films. The molecular weight decreased with the PCA content, while the dispersity was reduced. These phenomena and the interaction between polymer chains, ruled by the aromatic and aliphatic nature of the repetitive units, influence the final characteristics of the copolymers. In general, melting points, enthalpies of fusion, and breathability were reduced as PCA content is raised. Meanwhile water uptake capacity was kept very low and plasticity was improved. All samples were bio- and hemocompatible except PCL/PCA 2:1. Moreover, the presence of PCA in the copolymers considerably increases the antioxidant properties and can partially inhibit the growth of *E*. *coli*. Considering the above results, the copolymer with mole ratio 4:1 can be considered as a suitable biomaterial for skin regeneration in wound management.

## Supporting information

S1 Fig 1D and 2D spectra of PCL. PLC in THF-d8 NMR spectra: a) 1H b) 1H-1H COSY c) 1H-13C HSQC.
(PDF)

S2 Fig Cellular morphological analysis. Representative morphological images of HDFa cells incubated or not (control) with t PCL-PCA 1:0, 10:1, 8:1, 6:1, 4:1, 2:1 films for 72h.
(PDF)

S3 Fig Inhibition zone assay. Antibacterial effect of PCL-PCA films. *E*. *coli* growth in presence of PCL/PCA 1:0 (A), 6:1 (B), 4:1 (C), and 2:1 (D) for 24 and 72 hours.
(PDF)
