# Supplementary material for: Low molecular weight ε-caprolactone-p-coumaric acid copolymers as potential biomaterials for skin regeneration applications
Source: PLoS One. 2019 Apr 8;14(4):e0214956. doi: 10.1371/journal.pone.0214956 (PMC6453441; doi:10.1371/journal.pone.0214956)
Supplement: S1 Fig — PLC in THF-d8 NMR spectra: a) 1H b) 1H-1H COSY c) 1H-13C HSQC. (PDF) [file pone.0214956.s001.pdf]

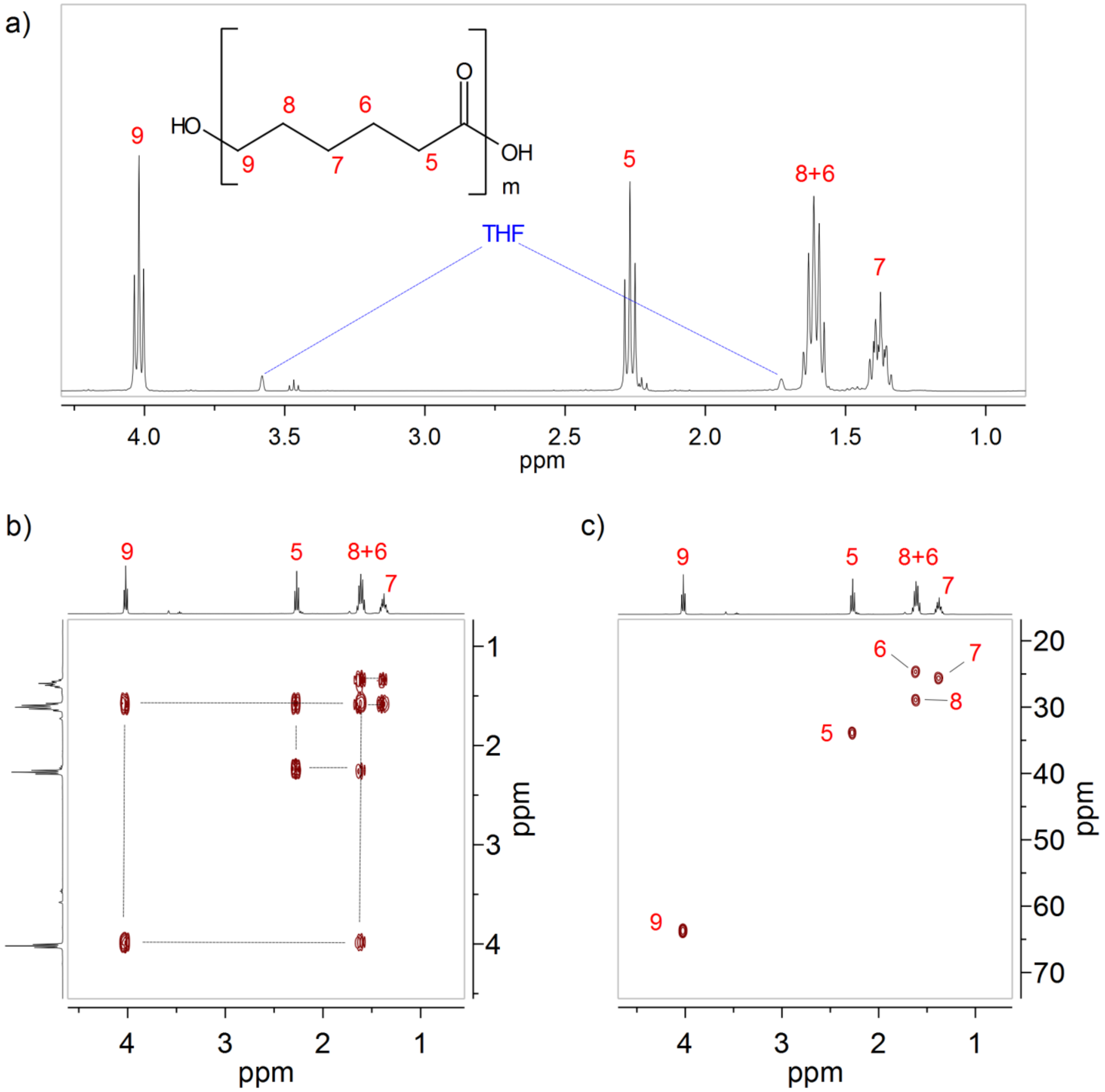

S1 Fig. 1D and 2D spectra of PCL. PLC in THF-d8 NMR spectra: a) 1H b) 1H-1H COSY c) 1H-13C HSQC.
