# Supplementary material for: Low molecular weight ε-caprolactone-p-coumaric acid copolymers as potential biomaterials for skin regeneration applications
Source: PLoS One. 2019 Apr 8;14(4):e0214956. doi: 10.1371/journal.pone.0214956 (PMC6453441; doi:10.1371/journal.pone.0214956)
Supplement: S2 Fig — Representative morphological images of HDFa cells incubated or not (control) with t PCL-PCA 1:0, 10:1, 8:1, 6:1, 4:1, 2:1 films for 72h. (PDF) [file pone.0214956.s002.pdf]

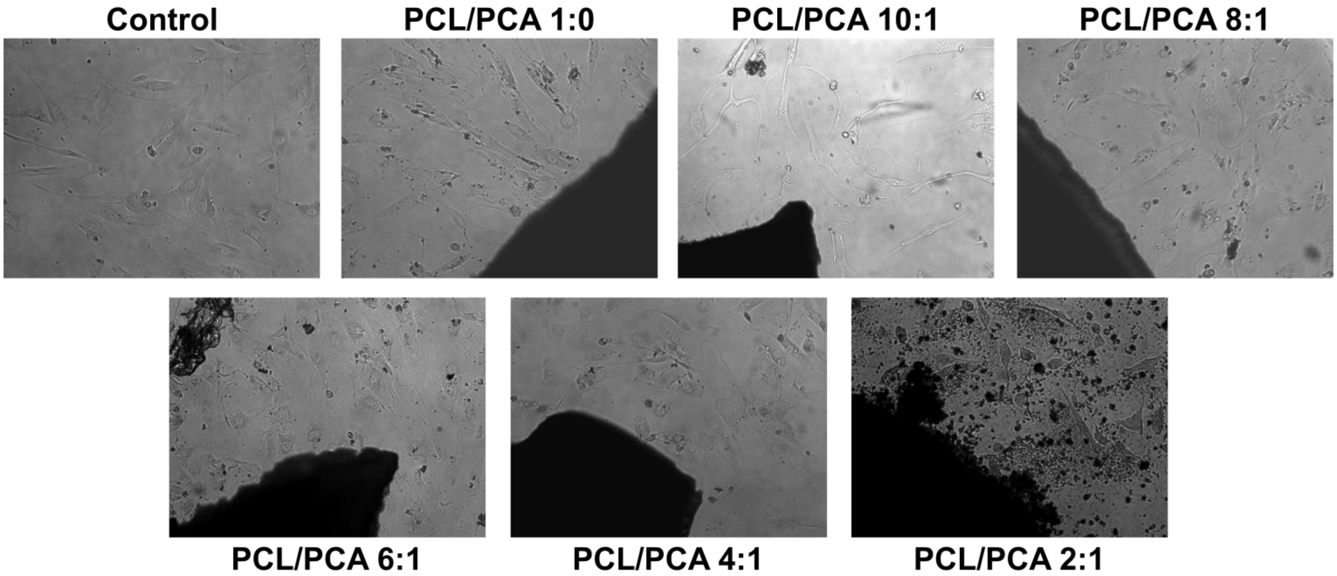

S2 Fig. Cellular morphological analysis. Representative morphological images of HDFa cells incubated or not (control) with t PCL-PCA 1:0, 10:1, 8:1, 6:1, 4:1, 2:1 films for 72h.
