# Supplementary material for: Low molecular weight ε-caprolactone-p-coumaric acid copolymers as potential biomaterials for skin regeneration applications
Source: PLoS One. 2019 Apr 8;14(4):e0214956. doi: 10.1371/journal.pone.0214956 (PMC6453441; doi:10.1371/journal.pone.0214956)
Supplement: S3 Fig — Antibacterial effect of PCL-PCA films. E. coli growth in presence of PCL/PCA 1:0 (A), 6:1 (B), 4:1 (C), and 2:1 (D) for 24 and 72 hours. (PDF) [file pone.0214956.s003.pdf]

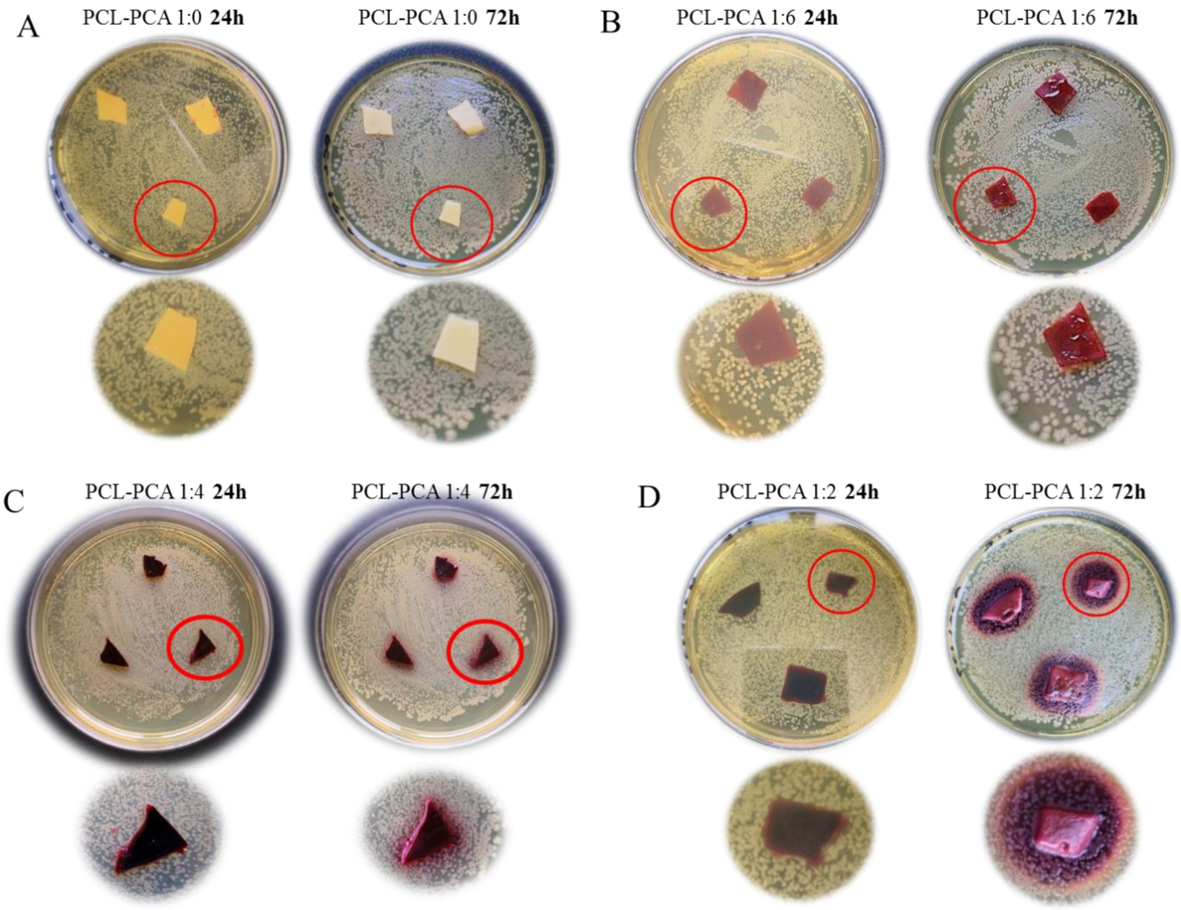

S3 Fig. Inhibition zone assay. Antibacterial effect of PCL-PCA films. *E. coli* growth in presence of PCL/PCA 1:0 (A), 6:1 (B), 4:1 (C), and 2:1 (D) for 24 and 72 hours.
